# Peroxidasin expression patterns predict fibrosis and kidney function decline in IgA nephropathy

**DOI:** 10.1007/s10157-026-02912-y

**Published:** 2026-07-27

**Authors:** Roberto Silva, Isabel Brandão, Tony J. Chen, Hans-Peter Marti, Bárbara Gomes, Bernardo Faria, Manuel Pestana, Jorge R. Almeida, Inês S. Alencastre, João Paulo Oliveira

**Affiliations:** 1 Department of Anatomic Pathology, São João Local Health Unit and University Hospital, Porto, Portugal; 2 Faculty of Medicine, University of Porto, Porto, Portugal; 3 i3S – Instituto de Investigação e Inovação em Saúde (i3S – Institute for Research and Innovation in Health), University of Porto, Porto, Portugal; 4 School of Medicine and Biomedical Sciences (ICBAS – Instituto de Ciências Biomédicas Abel Salazar), University of Porto, Porto, Portugal; 5 Department of Clinical Medicine, University of Bergen, Bergen, Norway; 6 Department of Medicine, Haukeland University Hospital, Bergen, Norway; 7 Department of Nephrology, São João Local Health Unit and University Hospital, Porto, Portugal; 8 Nephrology and Cardiovascular Risk Research Group, RISE-Health Research and Development Unit, Faculty of Medicine, University of Porto, Alameda Hernâni Monteiro, Porto, 4200-319 Portugal; 9 Department of Human Genetics, São João Local Health Unit and University Hospital, Alameda Hernâni Monteiro, Porto, 4200-319 Portugal

**Keywords:** Chronic kidney disease progression, Collagen type III, Compartment-specific expression: IgA Nephropathy, Interstitial fibrosis, Peroxidasin, Renal fibrogenesis

## Abstract

Peroxidasin (PXDN), a heme-containing peroxidase involved in basement membrane assembly, has been increasingly implicated in fibrotic processes, yet its role in kidney disease remains unclear. We therefore characterized PXDN expression in kidney biopsies from patients with IgA nephropathy (IgAN), a leading cause of CKD, and assessed its association with fibrosis. Immunofluorescence demonstrated widespread PXDN expression across renal compartments, with predominant localization in distal tubules, consistent with healthy tissue. In contrast, PXDN expression was reduced in proximal tubules, including non-atrophic segments, and markedly increased in the fibrotic interstitium. PXDN strongly correlated with collagen III deposition in sclerotic glomeruli and fibrotic interstitium, but not with collagen IV. Clinically, patients with impaired kidney function (eGFR < 60 ml/min/1.73 m²) exhibited higher PXDN expression in glomeruli, proximal tubules, and fibrotic interstitium, with glomerular PXDN inversely correlating with eGFR. These findings reveal compartment-specific alterations in PXDN expression in IgAN, associated with interstitial fibrosis, collagen III deposition, and declining kidney function. PXDN may participate in fibrotic remodeling processes and represents a potential therapeutic target, warranting further investigation in CKD progression.

**Supplementary Information:**

The online version contains supplementary material available at https://doi.org/10.1007/s10157-026-02912-y.

## Introduction

Peroxidasin (PXDN) is a multifunctional heme peroxidase that catalyzes the covalent crosslinking of collagen type IV (COL4) [1–3], a key structural component of basement membranes (BM), thereby contributing to extracellular matrix (ECM) organization and redox homeostasis [4]. In healthy kidney, PXDN is expressed across all compartments from early nephrogenesis, with prominent localization in distal tubules [5], suggesting functions beyond its enzymatic activity. Nevertheless, despite its evolutionary conservation and ubiquitous expression, the biological role of PXDN in kidney physiology and disease remains poorly defined.

Recent evidence has implicated PXDN in fibrotic processes [6], a central mechanism in chronic kidney disease (CKD) progression and a major cause of irreversible loss of function. This is of major clinical importance, as CKD affects approximately 10% of the global population and is projected to become the fifth leading cause of death by 2040 [7].

Immunoglobulin A nephropathy (IgAN), the most common primary glomerulonephritis worldwide, results in kidney failure (KF) in up to 50% of patients within two decades [8, 9]. It is characterized by mesangial IgA deposition, inflammation and progressive glomerular sclerosis and tubulointerstitial fibrosis. Owing to its well-defined histopathological criteria [10–12] and strong association between tubular atrophy, interstitial fibrosis, and clinical outcomes, IgAN provides a robust model to investigate mechanisms driving CKD progression.

In this exploratory study, we tested the hypothesis that PXDN may be involved in the pathogenesis of progressive kidney disease using IgAN as a model. To our knowledge, this is the first study to examine PXDN expression in a human kidney disease. Using formalin-fixed, paraffin-embedded (FFPE) kidney biopsies, we characterized PXDN expression in patients with IgAN and in histologically normal-appearing kidney tissue used as control. Within IgAN specimens, we further compared its distribution in fibrotic vs. non-fibrotic regions and assessed its association with established histopathological markers of interstitial fibrosis, namely Sirius Red staining and collagen type III (COL3) immunohistochemistry [13, 14].

## Materials and methods

### Ethical approval

This study was conducted in accordance with the Declaration of Helsinki. The research protocol was reviewed and approved by the Health Ethics Commission (reference number CE-04–2023) and Data Protection Officer of *São João* Local Heath Unit and University Hospital (HUSJ), Porto, Portugal, as well as by the Institutional Review Board of Haukeland University Hospital (HUH), University of Bergen (UiB), Bergen, Norway (REK vest 2013/553, including active patient consent for shipping biopsy sections abroad).

### IgA nephropathy kidney biopsies

Kidney biopsy samples from patients with IgAN were obtained from the archives of the Department of Anatomic Pathology, HUSJ. A total of 20 FFPE biopsies containing at least one intact glomerulus were included. The relevant demographic, clinical and kidney pathology features of the IgAN patient cohort are summarized in Table 1. For the planned histological and immunofluorescence analyses, 3-µm sections were prepared from each FFPE block and mounted on pseudonymized glass slides following standardized laboratory protocols. IgAN kidney biopsies were further stratified according to a semiquantitative histological assessment of glomerular IgA deposition, graded as +, ++, and +++.

**Table 1:** Demographic, clinical, laboratory and histopathological features of the IgA nephropathy patient cohort

Characteristics	
Sex: Female | Male; N (%)	9 (45) | 11 (55)
Age^✝^, years; median (IQR)	49.4 (45.2–57.9)
Creatinine^✝^, mg/dL; median (IQR)	1.7 (1.2–2.2)
eGFR^✝^, mL/min/1.73m^2^; median (IQR)	47 (29.2–75.2)
Proteinuria^✝^, g/day; median (IQR)	2.8 (1.5–4.5)
Presence of hematuria^✝^; N (%)	18 (90)
Number of segmented glomeruli, per biopsy; median (IQR)	3 (2–6.25)^⁑^
^*^Mesangial hypercellularity: M0 | M1; N (%)	14 (70) | 6 (30)
^*^Endocapillary hypercellularity: E0 | E1; N (%)	20 (100) | 0 (0)
^*^Segmental sclerosis: S0 | S1; N (%)	3 (15) | 17 (85)
^*^Tubular atrophy/interstitial fibrosis: T0 | T1 | T2; N (%)	8 (40) | 9 (45) | 3 (15)
^*^Crescents: C0 | C1 | C2; N (%)	17 (85) | 3 (15) | 0 (0)

^✝^At the time of kidney biopsy. ^*^Pathologic features of the Oxford Classification of IgA Nephropathy (MEST-C score) [9].

eGFR, estimated glomerular filtration rate; IQR, interquartile range; N, number

^⁑^When plotted against the number of glomeruli analyzed in biopsies with ≥ 2 segmented glomeruli (*n* = 17), the coefficient of variation of glomerular PXDN expression remained consistently below 10%, indicating low intra-biopsy variability and suggesting that increasing the number of segmented glomeruli does not substantially affect measurement variability (Supplementary Fig. 2)

**Table 2:** Demographic, clinical, laboratory and histopathological features of the IgA nephropathy patient cohort according to estimated glomerular filtration rate stratification

Characteristics	eGFR ≥ 60 mL/min/1.73m^2^	eGFR < 60 mL/min/1.73m^2^	*P*-value
Sex: Female | Male; N (%)	2 (25) | 6 (75)	7 (58) | 5 (42)	0.197^1^
Age^✝^, years; median (IQR)	52.7 (41.5–62.4)	47.9 (45.2–56.3)	0.847^2^
Creatinine^✝^, mg/dL; median (IQR)	1.1 (0.7–1.2)	2.1 (1.7–3.6)	**< 0.0001** ^3^
eGFR^✝^, mL/min/1.73m^2^; median (IQR)	77 (71.5–104.3)	30 (15.2–39.8)	**< 0.0001** ^2^
Proteinuria^✝^, g/day; median (IQR)	1.9 (0.9–4.5)	3.1 (2.3–4.5)	0.448^3^
Presence of hematuria^✝^; N (%)	6 (75)	12 (100)	0.147^1^
^*^Mesangial hypercellularity: M0 | M1; N (%)	5 (62.5) | 3 (37.5)	9 (75) | 3 (25)	0.642^1^
^*^Endocapillary hypercellularity: E0 | E1; N (%)	8 (100) | 0 (0)	12 (100) | 0 (0)	> 0.999^1^
^*^Segmental sclerosis: S0 | S1; N (%)	1 (12.5) | 7 (87.5)	2 (17) | 10 (83)	> 0.999^1^
^*^Tubular atrophy/interstitial fibrosis: T0 | T1 | T2; N (%)	4 (50) | 4 (50) | 0 (0)	4 (33) | 5 (43) | 3 (25)	0.300^4^
^*^Crescents: C0 | C1 | C2; N (%)	7 (87.5) | 1 (12.5) | 0 (0)	10 (83) | 2 (17) | 0 (0)	> 0.999^1^

^✝^At the time of kidney biopsy. ^*^Pathologic features of the Oxford Classification of IgA Nephropathy (MEST-C score) [9]

The bold text highlights statistical significance (p<0.05)

eGFR estimated glomerular filtration rate; IQR interquartile range; N number

^1^Fisher’s exact test. ^2^Unpaired t-test. ^3^Mann-whitney. ^4^Chi-square test

### Clinical stratification of IgA nephropathy

To explore a possible relationship between PXDN expression and the severity of kidney disease, patients diagnosed with IgAN were grouped into two categories, based on their CKD stage at the time of biopsy. CKD stages were defined according to the “Kidney Disease: Improving Global Outcomes” (KDIGO) 2024 guidelines [15], using the creatinine-based estimated glomerular filtration rate (eGFR), calculated by the CKD Epidemiology Collaboration (CKD-EPI) 2009 formula [16, 17]. PXDN expression was compared between patients with CKD stage ≤ 2 (eGFR ≥ 60 mL/min/1.73 m²) and patients with CKD stage ≥ 3 (eGFR < 60 mL/min/1.73 m²). Table 2 summarizes comparisons of relevant demographic, clinical, and kidney pathology characteristics between the two groups.

### Sources of histologically normal-appearing human kidney tissue

Since no published data exist on PXDN expression in FFPE human kidney tissue, and because ischemic intervals prior to fixation differ substantially between nephrectomy and percutaneous biopsy specimens [18], we evaluated two sources of histologically normal kidney tissue as potential controls: (*i*) percutaneous kidney biopsy samples obtained for clinical indications but ultimately classified as histologically normal (*n* = 7), provided by the Department of Medicine, HUH-UiB; and (*ii*) peritumoral nephrectomy specimens from patients with renal cell carcinoma (*n* = 7) or non-malignant kidney conditions (*n* = 3), provided by the Department of Anatomic Pathology, HUSJ. (See Table 3 for information on the demographic and clinical characteristics of the individuals from whom histologically normal kidney tissue samples were obtained, and comparison with those of the IgAN cohort). In cancer nephrectomies, tissue was collected at least 1 cm from the tumor margin. For histology and immunofluorescence analyses, 3-µm sections containing at least one intact glomerulus were prepared from each FFPE block and mounted on pseudonymized glass slides using standard laboratory protocols.

**Table 3:** Kidney tissue sources and demographic and clinical characteristics of IgA nephropathy patients and controls

Kidney tissue source	Reason for nephrectomy/kidney biopsy	*N*	Gender [M | F]	Median age [range], years
Peritumoral normal-appearing tissue from nephrectomy specimens^¶^	Renal cell carcinoma	7	[7 M]	50.5 [31–69] ^*a*^
	Angiomyolipoma	1	[1 F]	
	Blunt kidney trauma	1	[1 M]	
	Early acute allograft renal artery thrombosis	1	[1 M]	
Biopsy samples classified as histologically normal^†^	Clinical indication: transient renal dysfunction and/or urinary abnormalities	7	[5 M | 2 F]	47 [22–58] ^*b*^
Biopsy samples diagnosed as IgA nephropathy^¶^	Diagnostic kidney biopsy	20	[11 M | 9 F]	50 [37–74] ^*c*^

^¶^Provided by the Department of Anatomic Pathology, *São João* Local Health Unit and University Hospital, Porto, Portugal; ^†^provided by Haukeland University Hospital, University of Bergen, Bergen, Norway.

F female; M male; N number.

*P*-values for age differences (one-way ANOVA and Tukey’s multiple comparison test): *p =* 0.2331 when compared ^*a*^ vs. ^*b*^; *p =* 0.9956 when compared ^*a*^ vs. ^*c*^; *p =* 0.1881 when compared ^*b*^ vs. ^*c*^

### Processing of the kidney tissue specimens for brightfield microscopy and manual segmentation

For brightfield microscopy, unstained and periodic acid–Schiff (PAS)-stained slides were prepared from sequential sections of each selected FFPE block, at room temperature, following standard histopathology procedures. Briefly, tissue sections were deparaffinized in fresh xylene (3 × 10 min) and rehydrated through a graded ethanol series (100%, 96%, 70%, and 50%, 10 min each) before immersion in double-distilled water. For PAS staining, sections were oxidized in 0.5% periodic acid for 5 min, incubated with Schiff’s Reagent for 30 min, and counterstained with hematoxylin for 3 min. Unstained slides were used for manual segmentation of kidney tissue compartments, while PAS-stained slides served as a topographical reference to guide semantic segmentation and to verify the histomorphology of both healthy and diseased regions.

### Processing of the kidney tissue specimens for indirect immunofluorescence analysis

After deparaffinization and rehydration, slides prepared for immunodetection were subjected to antigen retrieval in sodium citrate solution (pH 6.0) at 95 °C for 20 min, followed by cooling at room temperature for 20 min. Slides were then washed in phosphate-buffered saline (PBS, pH 7.4) and incubated with blocking/permeabilization buffer (10% normal horse serum and 0.1% Triton X-100 in PBS) for 60 min at room temperature. Following blocking, slides were incubated overnight at 4 °C with primary antibodies diluted in blocking buffer. After washing in PBS, slides were incubated for 1 h at room temperature with the corresponding secondary antibodies, also diluted in blocking buffer. Nuclei were counterstained with 4′,6-diamidino-2-phenylindole (DAPI) in PBS. After thorough PBS washes, slides were mounted with ProLong™ Gold Diamond Antifade Mountant (Thermo Fisher Scientific, Waltham, MA, USA; catalog # P36965), allowed to autopolymerize overnight at room temperature, and stored in the dark at 4 °C until analysis.

### Primary and secondary antibodies

Samples were double-stained with two sets of primary antibodies: (*i*) a validated anti-PXDN (Rabbit; Abbexa, Cambridge, UK; catalog # abx101906) [19, 20] combined with anti-Cluster of Differentiation 15 (CD15) (Mouse; Ventana/Roche, Roche Diagnostics, Rotkreuz, Switzerland; catalog # 760–2504); and (*ii*) anti-Collagen type III alpha-1 (Rabbit; ProteinTech, Manchester, UK; catalog # 22734-1-AP) combined with anti-Collagen type IV alpha-1 (Mouse; GeneTex, California, USA; catalog # GTX26311).

Fluorophore-conjugated secondary antibodies were used to detect the corresponding primary antibodies: Donkey Anti-Rabbit IgG (H + L), Highly Cross-Adsorbed, Alexa Fluor™ Plus 647 (Invitrogen, Thermo Fisher Scientific, Waltham, MA, USA; catalog # A32795) for rabbit primaries, and Mouse IgG (H + L), Highly Cross-Adsorbed, Alexa Fluor™ Plus 568 (Invitrogen, Thermo Fisher Scientific; catalog # A10037) for mouse primaries.

### Whole slide imaging, digital image analysis, manual segmentation and virtual microscopy

Glass slides processed for brightfield or fluorescence microscopy were digitized at high resolution using a Nanozoomer S60 Digital Slide Scanner (Hamamatsu Photonics, Hamamatsu, Japan). Brightfield images were manually segmented into glomeruli, tubules, vessels, and interstitium using the open-source QuPath software (https://qupath.github.io/), as illustrated in Supplementary Fig. 1. Segmentation was performed by one of two qualified annotators (I.B., I.S.A.), both with extensive prior experience in manual segmentation, working under the training and supervision of an expert nephropathologist (R.S.). All annotations underwent visual review and validation by the supervising nephropathologist. Both annotators and the supervising nephropathologist were blinded to patients’ CKD stage classification. Regions classified as damaged were identified according to established histopathological features, including glomerulosclerosis, tubular atrophy, interstitial fibrosis, inflammatory infiltrates, loss of brush border, tubular simplification, epithelial flattening, and luminal dilatation.

Proximal and distal tubules were distinguished based on CD15 immunolabelling. In IgAN samples, segmented structures were further classified according to the presence or absence of histopathological lesions, including sclerotic glomeruli (> 50% sclerosis), tubular atrophy, interstitial fibrosis, and inflammatory infiltrates. Interstitial fibrosis was defined according to standard histopathological criteria [13, 14]. To compare PXDN expression in fibrotic and non-fibrotic regions within the same biopsy, fibrotic areas were identified by Sirius Red staining, a well-established histochemical marker of fibrillar collagen deposition (predominantly collagen I and III). Fluorescence intensity was quantified in regions of interest defined in QuPath using ImageJ/Fiji [21] following Rolling Ball Background Subtraction.

### Kidney segmentation reproducibility

To assess inter-annotator reproducibility, the same regions of a random subset of 79 image tiles (512 × 512 pixels) from the study dataset were independently segmented by both annotators using the same workflow. Agreement was evaluated using the Dice Similarity Coefficient (DSC) and Intersection-over-Union (IoU) [22]. High agreement was observed across all kidney compartments (Supplementary Table 1), supporting the reproducibility of the segmentation approach.

### Data analyses

Normally distributed data from histologically normal kidney tissue specimens were compared using one-way analysis of variance (ANOVA) followed by Tukey’s post-hoc test for multiple comparisons, or unpaired t-tests where appropriate. Normality was assessed using the Shapiro–Wilk test. For IgAN samples, non-parametric analyses were performed using the Kruskal–Wallis test with Dunn’s post-hoc multiple comparisons test and unpaired Mann–Whitney tests, as appropriate. Correlations were assessed using two-tailed Pearson correlation for normally distributed variables and two-tailed Spearman rank correlation for non-normally distributed variables. Clinical variables were compared using Fisher’s exact test or the chi-square test for categorical data, and unpaired t-tests or Mann–Whitney tests for continuous data, as appropriate. Statistical analyses were performed with GraphPad Prism version 8.0.2 for Windows (GraphPad Software, Boston, MA, USA). A two-sided p value < 0.05 was considered statistically significant.

## Results

### Demographic comparability and measurement reproducibility

Kidney tissue samples from patients with IgA nephropathy (IgAN) and controls showed comparable demographic characteristics (Table 2). Median age was similar across groups, with no statistically significant differences, and all cohorts displayed a predominance of males.

Quantitative image analysis of glomerular PXDN expression demonstrated low coefficients of variation, which did not significantly correlate with the number of segmented glomeruli (Supplementary Fig. 2). This indicates that the measurements were not biased by differences in tissue sampling or glomerular representation across specimens.

### Histologically normal-appearing kidney tissue controls: nephrectomy specimens vs. biopsy samples

**Fig. 1:**
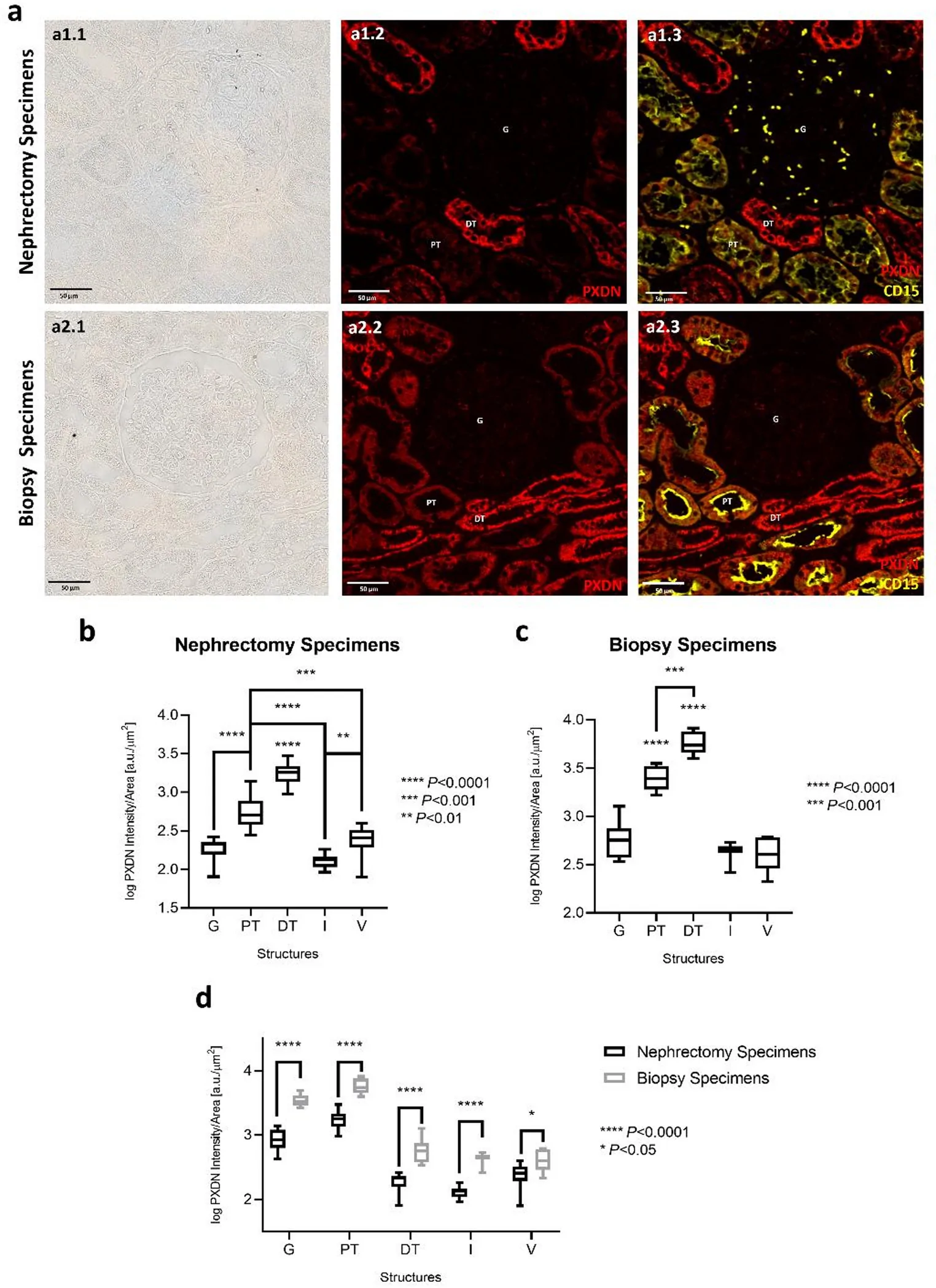
Representative photomicrographs and quantitative comparison of peroxidasin (PXDN) expression across histological compartments in histologically normal-appearing kidney tissue, obtained from clinical biopsies (*n* = 7; median age = 47 years) or non-lesioned peritumoral nephrectomy areas (*n* = 10; median age = 50.5 years), . Panel **a** shows representative sections of PXDN staining of nephrectomy [a1.2] and biopsy specimens [a2.2] and corresponding brightfield [a1.1, a2.1] and overlay with CD15 staining [a1.3, a2.3]. Statistical analyses were performed with GraphPad Prism^®^ software using one-way ANOVA and Tukey’s multiple comparisons test (**b**, **c**) and unpaired t-test (**d**). Intensity/area values were obtained by dividing the integrated density by the total area of the compartment. Additional abbreviations ‒ a.u. arbitrary units; G glomeruli; PT proximal tubules; DT distal tubules; I interstitium; V vessels

To define suitable controls for PXDN expression in IgAN biopsies, we compared histologically normal-appearing kidney parenchyma obtained from cancer nephrectomy specimens and from kidney biopsies of patients presenting with transient renal dysfunction and/or urinary abnormalities. PXDN expression patterns across kidney structures were similar in both sources (Fig. 1a), with the highest expression in distal tubules (Fig. 1b-c) as previously observed [5]; however, fluorescence intensity per unit area was significantly higher in biopsy samples for all structures analyzed (Fig. 1d), indicating that nephrectomy tissue may not be an optimal control (see discussion). Therefore, all subsequent analyses used biopsy-derived kidney tissue with non-significant histological changes, as the control group for comparison with IgA nephropathy samples.

### Peroxidasin expression in IgA nephropathy is compartment-specific

**Fig. 2:**
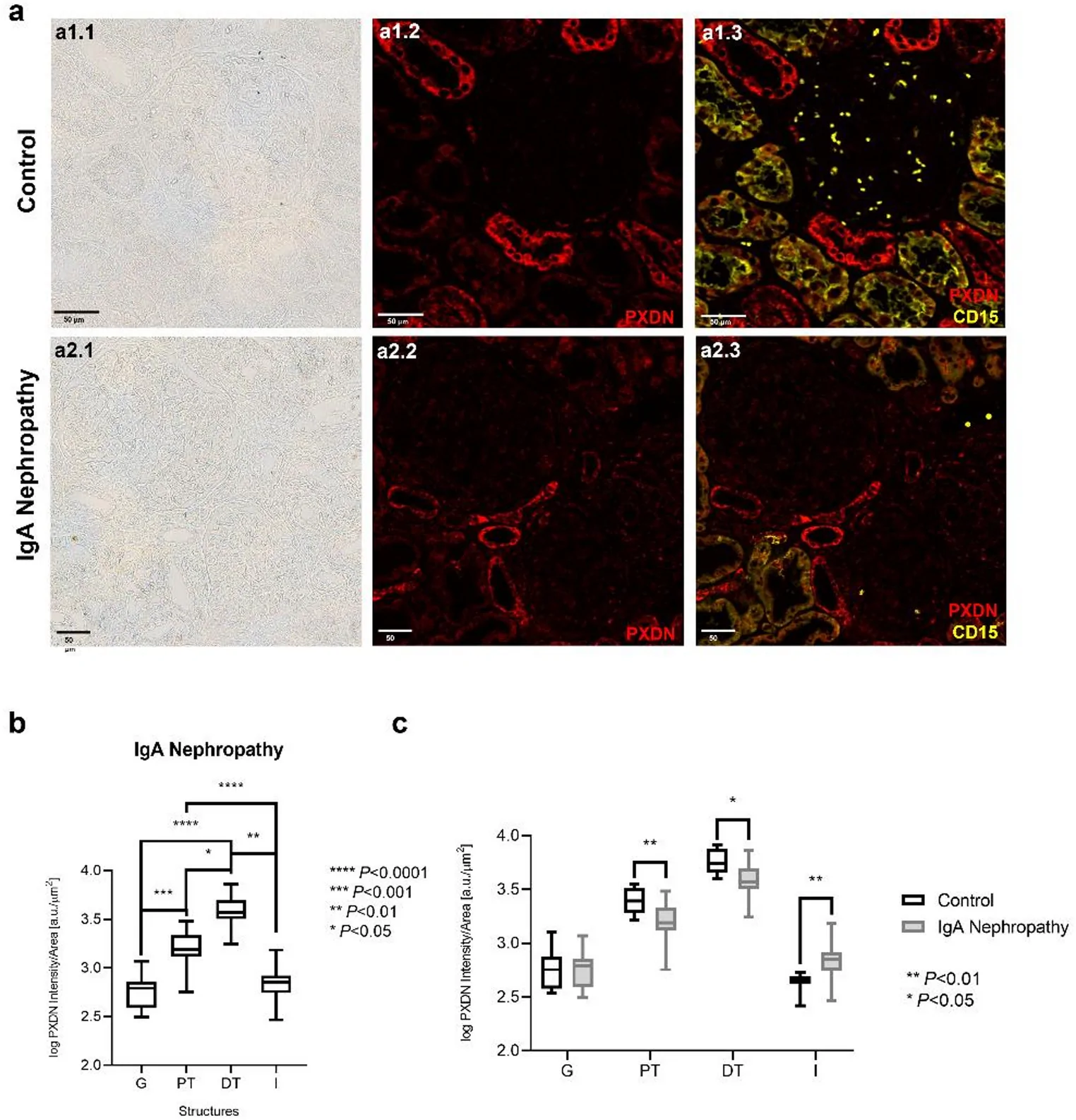
Representative photomicrographs and quantitative comparison of PXDN expression across histological compartments in IgA nephropathy (IgAN) kidney biopsy specimens (*n* = 20; median age = 49.4 years), and between IgAN and healthy kidney biopsy specimens (*n* = 7; median age = 47 years). Panel **a** shows representative PXDN staining sections of control biopsy specimens (a1.2) and IgAN kidney biopsy (a2.2) and corresponding brightfield [a1.1, a2.1] and overlay with CD15 staining [a1.3, a2.3]. Statistical analyses were performed with GraphPad Prism^®^ software using Kruskal-Wallis and Dunn’s multiple comparisons test (**b**) and unpaired Mann-Whitney (**c**). Intensity/area values were obtained by dividing the integrated density by the total area of the compartment. Additional abbreviations ‒ a.u. arbitrary units; G glomeruli; PT proximal tubules; DT distal tubules; I interstitium; IgA immunoglobulin A

In both IgAN and normal-appearing kidney tissue, PXDN displayed a punctate cytoplasmic distribution (Fig. 2a), with higher expression in distal tubules (Fig. 2b). Compared with controls, PXDN expression was significantly reduced in proximal and distal tubules (Fig. 2c; proximal tubules *p* < 0.01, distal tubules *p* < 0.05), while interstitial expression was increased (Fig. 2c; *p* < 0.01). Glomerular PXDN expression did not differ significantly between groups (Fig. 2c).

**Fig. 3:**
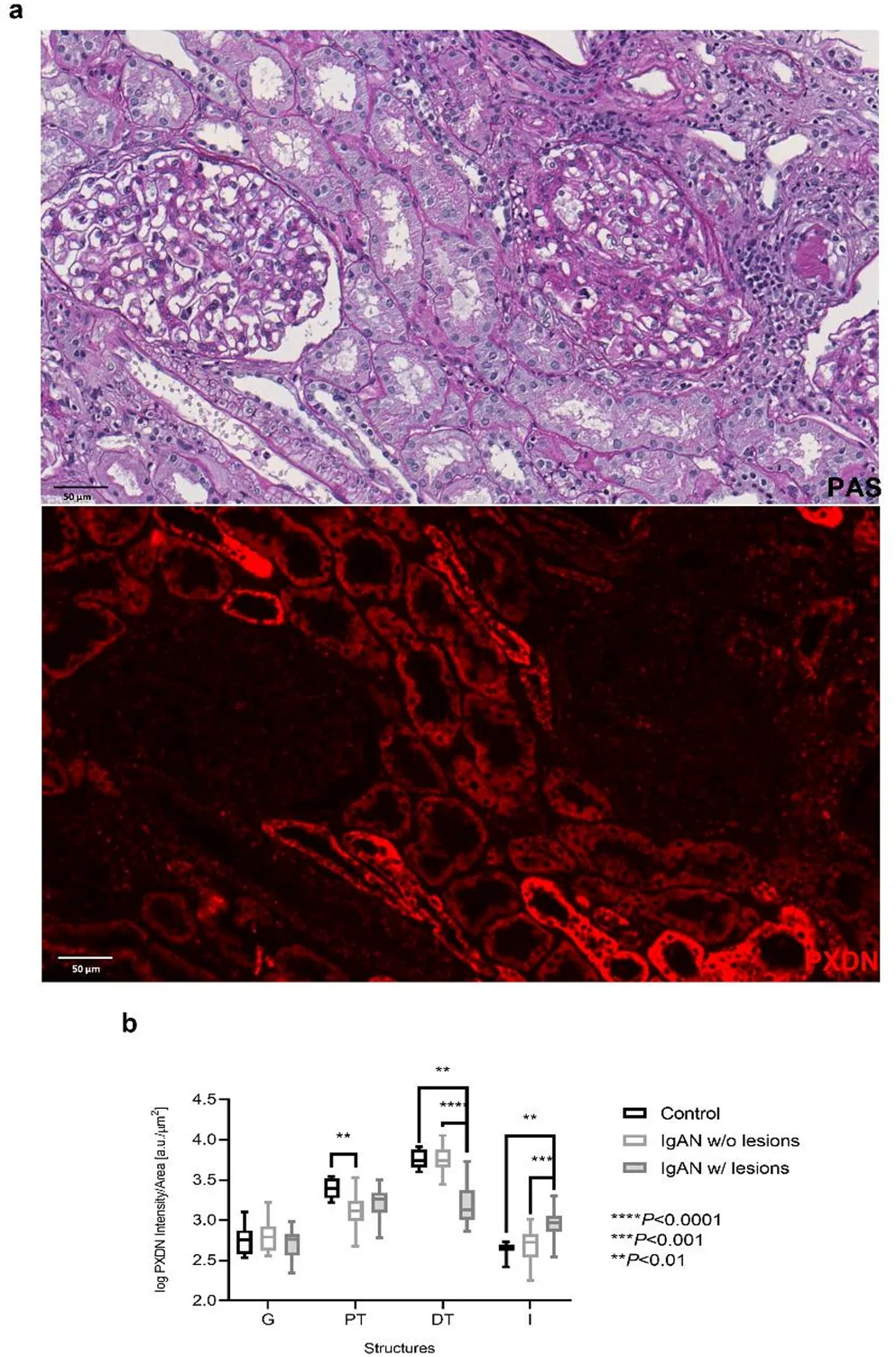
Representative photomicrographs and quantitative comparison of peroxidasin (PXDN) expression across histological compartments in areas with (w/) or without (w/o) sclerotic/fibrotic lesions from IgA nephropathy (IgAN) kidney biopsy specimens (*n* = 20; median age = 49.4 years), and healthy kidney biopsy specimens (*n* = 7; median age = 47 years). Panel **a** shows a periodic acid-Schiff (PAS)-stained histological section of an IgAN kidney biopsy (upper section) and the corresponding PXDN-stained image (lower section). Statistical analyses were performed with GraphPad Prism^®^ software using Kruskal-Wallis and Dunn’s multiple comparisons test within each compartment. Intensity/area values were obtained by dividing the integrated density by the total area of the compartment. Additional abbreviations ‒ a.u. arbitrary units; G glomeruli; PT proximal tubules; DT distal tubules; I interstitium; V vessels; IgA immunoglobulin A

PXDN expression was further compared in regions with and without sclerotic/fibrotic lesions within each histological compartment of IgAN biopsies (Fig. 3a). In proximal tubules, PXDN expression was reduced in both atrophic and non-atrophic tubules relative to controls (Fig. 3b; *p* < 0.01), with no significant difference between the two subgroups. In distal tubules, decreased PXDN expression was confined to atrophic tubules (Fig. 3b; *p* < 0.01 vs. controls; *p* < 0.001 vs. IgAN without lesions), whereas non-atrophic distal tubules showed levels comparable to controls. In the interstitium, PXDN expression was significantly higher in fibrotic areas than in both histologically normal-appearing kidney tissue (Fig. 3b; *p* < 0.01) and non-fibrotic IgAN regions (Fig. 3; *p* < 0.001). In glomeruli, PXDN expression did not differ significantly between IgAN and controls, regardless of the presence or absence of sclerosis (Fig. 3b).

### Peroxidasin expression correlates with collagen type III but not with collagen type IV in sclerotic glomeruli and fibrotic interstitium

**Fig. 4:**
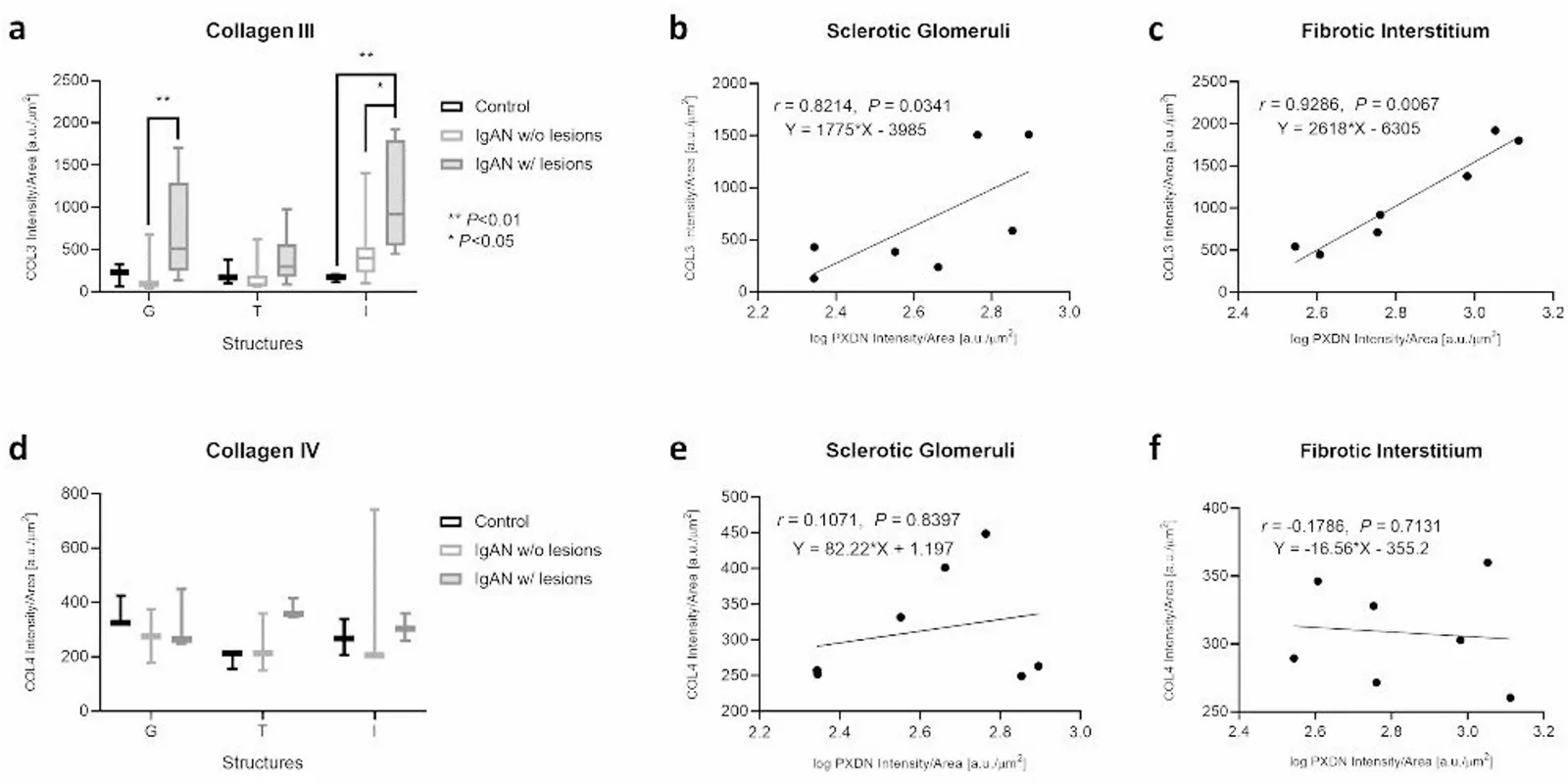
Quantitative comparison of Collagen type III and Collagen type IV expression across histological compartments in healthy kidney biopsy specimens (*n* = 7; median age = 47 years), and in areas with (w/) or without (w/o) sclerotic/fibrotic lesions from IgA nephropathy (IgAN) kidney biopsy specimens (*n* = 7; median age = 52.25 years). Statistical analyses were performed using GraphPad Prism^®^ software. Within-compartment comparisons [a, d] were conducted using the Kruskal–Wallis test with Dunn’s multiple-comparisons test, and correlations were assessed using Spearman’s rank correlation [b, c, e, f]. Intensity/area values were obtained by dividing the integrated density by the total area of the compartment. Additional abbreviations ‒ a.u. arbitrary units; G glomeruli; T tubules; I interstitium; IgA immunoglobulin A

COL3 expression, used as a marker of renal interstitial fibrosis, was significantly increased in sclerotic glomeruli and fibrotic interstitium compared with both control biopsies and histologically preserved regions of IgAN biopsies (Fig. 4a; *p* < 0.01 vs. controls; *p* < 0.05 in interstitium). PXDN expression correlated strongly with COL3 in sclerotic glomeruli (Fig. 4b; *r* = 0.8214, *p* = 0.0341) and in the fibrotic interstitium (Fig. 4c; *r* = 0.9286, *p* = 0.006). These areas also exhibited significantly greater Sirius Red staining than non-lesioned areas (Supplementary Fig. 4), consistent with increased fibrillar collagen deposition within fibrotic tissue. Contrastingly, no correlations were observed between PXDN and COL4 expression in any kidney compartment (Fig. 4d, e,f; Supplementary Table 2; representative photomicrographs in Supplementary Fig. 3).

### Peroxidasin expression is increased in glomeruli, proximal tubules, and fibrotic interstitium of IgAN patients with reduced kidney function

**Fig. 5:**
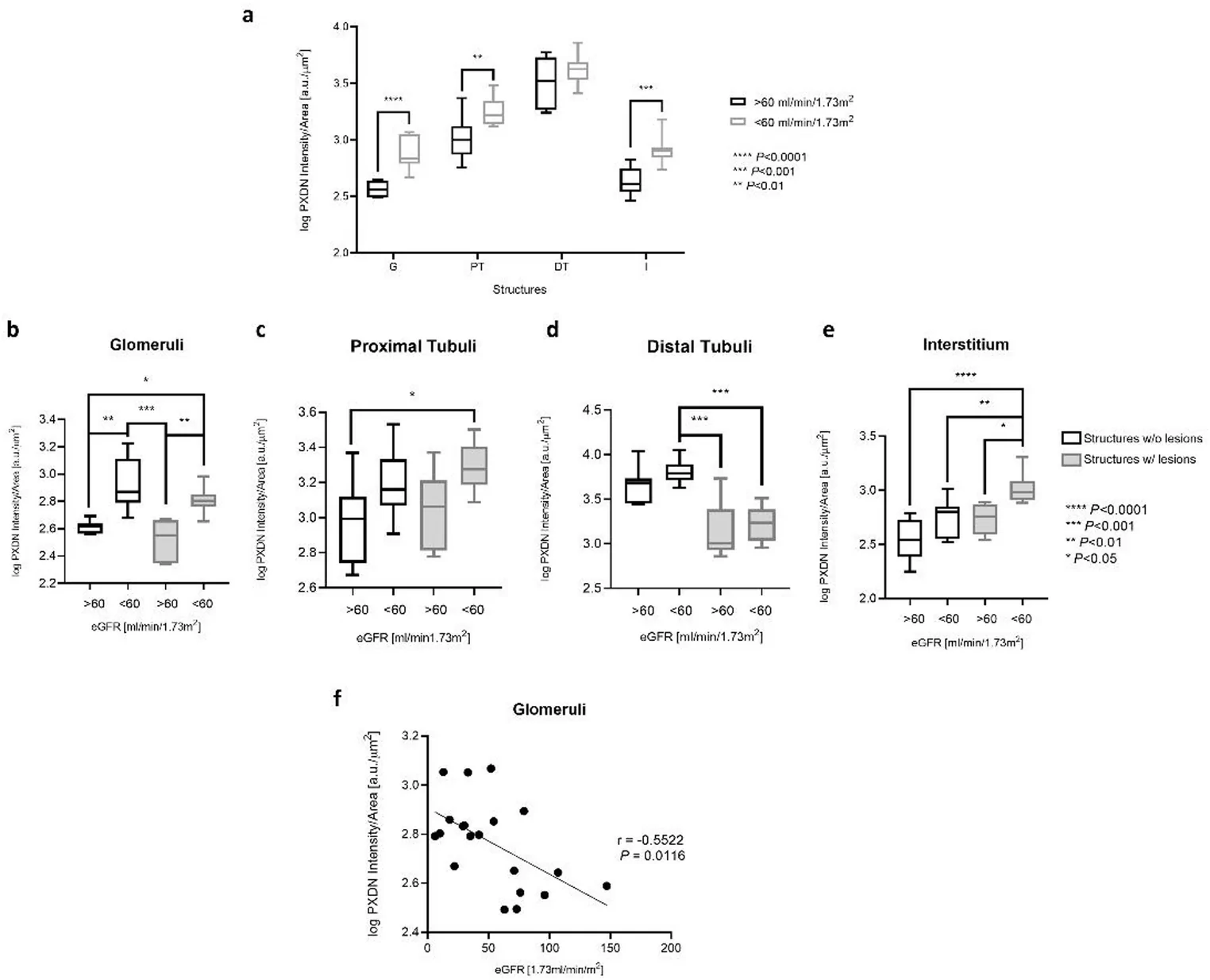
Quantitative comparison of peroxidasin (PXDN) expression across histological compartments in IgA nephropathy (IgAN) kidney biopsy specimens (*n* = 20; median age = 49.4 years), stratified by estimated glomerular filtration rate (eGFR) above (*n* = 8) or below (*n* = 12) 60 ml/min/1.73m^2^, in the whole sections (**a**) as well as in areas with (w/) or without (w/o) sclerotic/fibrotic lesions within the same sections (**b**–**e**). eGFR was computed from serum creatinine level at the time of biopsy, with values <60 ml/min/1.73m^2^ corresponding to chronic kidney disease (CKD) stages ≥3, and values ≥60 ml/min/1.73m^2^ corresponding to CKD stage ≤2. All statistical analyses were performed with GraphPad Prism^®^ software using unpaired Mann-Whitney for PXDN expression per histological compartment in the whole sections (**a**), and Kruskal-Wallis and Dunn’s multiple comparisons test for PXDN expression in areas w/or w/o sclerotic/fibrotic lesions, in the glomeruli (**b**), proximal tubules (**c**), distal tubules (**d**) and interstitium (**e**). In (**f**), r and p values were computed by two-tailed Pearson correlation. Intensity/area values were obtained by dividing the integrated density by the total area of the compartment. Additional abbreviations ‒ a.u. arbitrary units; IgA immunoglobulin A

IgAN patients with reduced eGFR (< 60 mL/min/1.73 m²) showed higher glomerular PXDN expression, independent of sclerosis (Fig. 5a-b; *p* < 0.0001, *p* < 0.01), with a significant negative correlation with eGFR (Fig. 5f; *r*= −0.5522; *p* = 0.0116). PXDN expression in proximal tubules tended to be higher in patients with reduced eGFR, reaching statistical significance only in atrophic proximal tubules relative to those from patients with preserved kidney function (Fig. 5c; *p* < 0.05). In distal tubules, PXDN expression was decreased in the presence of tubular atrophy but showed no association with eGFR (Fig. 5d). In the interstitium, PXDN expression was significantly higher in fibrotic regions of patients with reduced eGFR compared with non-lesioned interstitium from both eGFR groups, as well as with fibrotic interstitium from patients with preserved function (Fig. 5e; *p* < 0.001 vs. controls; *p* < 0.01 vs. non-lesioned IgAN; *p* < 0.05 vs. interstitium with lesions, eGFR > 60).

### Glomerular IgA deposition does not influence peroxidasin expression

No association was observed between glomerular IgA deposition and PXDN expression in any kidney compartment of IgAN biopsies (Supplementary Fig. 5).

## Discussion

PXDN is a heme-containing peroxidase with a central role in ECM remodeling, primarily through the crosslinking of the COL4 network in BM. However, its specific contribution to kidney pathology—and particularly to renal fibrosis in IgAN—has not been explored until now.

This study demonstrates significant alterations in PXDN expression patterns in IgAN kidney biopsies compared with histologically normal-appearing kidney tissue, supporting a potential role in disease pathogenesis. PXDN expression was reduced in proximal and distal tubules but increased in fibrotic interstitium and glomeruli. This compartment-specific distribution suggests that PXDN may play distinct roles in epithelial homeostasis and fibrotic remodeling during IgAN progression.

IgAN is the most common primary glomerulonephritis worldwide and a major contributor to CKD and KF [9]. While the disease is initiated by IgA deposition in glomeruli, driving inflammation and injury, the subsequent development of tubular atrophy and interstitial fibrosis is a major determinant of progression to KF. The specificity of IgAN diagnostic criteria, combined with the strong prognostic value of tubulointerstitial fibrosis, makes this disease a suitable model to investigate the potential role of PXDN in progressive CKD [10–12].

A critical methodological aspect of our study was the selection of appropriate controls. Although the relative distribution of PXDN across kidney compartments was consistent between histologically normal-appearing nephrectomy specimens and biopsies, overall fluorescence intensity was consistently higher (≈ 20%) in biopsies, suggesting that nephrectomy tissue underestimated baseline expression. This likely reflects the substantially longer ischemic intervals resulting from delayed fixation and processing of nephrectomy tissue [18] and/or possible paracrine effects of the respective tumors [23]. We therefore used biopsy-derived tissue as the reference for all comparative analyses, ensuring a more reliable baseline for interpreting compartment-specific changes in IgAN.

The decreased tubular expression of PXDN may indicate altered BM homeostasis and epithelial remodeling in diseased kidneys. Notably, the pattern differed between nephron segments: proximal tubules showed diffuse reduction, even in non-atrophic regions, whereas distal tubules displayed preserved PXDN expression except in overtly atrophic segments. These findings parallel single-cell transcriptomic data [24] which revealed that proximal tubules exhibit profound heterogeneity in injury states and readily adopt maladaptive phenotypes—particularly VCAM1 + failed-repair proximal tubular cells (FR-PTCs)—even when morphologically intact. In contrast, distal nephron segments (thick ascending limb, distal convoluted tubule, connecting tubule) displayed more stable epithelial profiles until atrophy became evident [25]. Although markers of tubular injury were not assessed in the present study, the distinct PXDN expression patterns across nephron segments may reflect differences in their response to pathological remodeling processes.

Conversely, the increased PXDN expression in the interstitium, especially within fibrotic areas, is consistent with its role in ECM organization and remodeling [4, 6]. Fibrosis, driven by excessive ECM deposition, is a central determinant of CKD progression [26–28]. The strong correlation between PXDN and COL3 deposition in sclerotic glomeruli and fibrotic interstitium provides the first evidence of an association between PXDN expression and fibrotic changes in IgAN. By contrast, COL4 expression did not differ between histologically normal-appearing kidney tissue and IgAN, regardless of sclerosis or fibrosis. These findings raise the possibility that PXDN functions may extend beyond its canonical role in BM assembly and may participate in maladaptive ECM remodeling during kidney fibrosis.

This interpretation is further supported by recent transcriptomic studies identifying proximal tubules as important contributors to tubulointerstitial fibrosis through interactions with fibroblasts [25, 28], with FR-PTCs promoting fibroblast-to-myofibroblast transition and excessive ECM deposition. Our compartment-specific PXDN signature—diffuse loss in proximal tubules, restricted reduction in distal tubules, and increased expression in fibrotic interstitium—appears broadly consistent with this epithelial–fibroblast–ECM axis. However, because the present study did not directly assess markers of tubular injury or repair pathways, any mechanistic relationship between PXDN expression and these processes remains speculative. PXDN may potentially contribute to the stabilization or organization of aberrantly deposited ECM components, although this hypothesis requires experimental validation.

When patients were stratified by CKD stage, glomerular PXDN expression was significantly higher in those with reduced kidney function (eGFR < 60 mL/min/1.73 m²), independent of sclerosis. The strong negative correlation between glomerular PXDN expression and kidney function suggests that PXDN upregulation reflects overall disease burden rather than specific histopathological lesions, underscoring its potential relevance as a biomarker of disease progression. Similarly, proximal tubular PXDN expression was higher in fibrotic regions of patients with impaired kidney function, further supporting an association between altered PXDN expression patterns and progressive tubulointerstitial remodeling.

Taken together, these findings suggest that PXDN expression may reflect differential responses across kidney compartments and may be associated with profibrotic remodeling accompanying CKD progression. PXDN may therefore represent both a candidate biomarker and a potential participant in pathological ECM remodeling, although functional studies are required to determine whether it plays a causal role.

This study has several limitations. First, the IgAN cohort size was modest and derived from a single center, which may limit generalizability. Second, the cross-sectional design precludes causal inferences between PXDN expression and disease progression. Third, analyses were restricted to FFPE material, preventing functional assays and limiting dynamic localization studies, particularly because insufficient material was available for RT-PCR validation at the mRNA level. Fourth, markers of tubular injury were not assessed; therefore, the observed alterations in tubular PXDN expression cannot be directly interpreted as evidence of tubular injury. Fifth, COL3 was used as an established marker of renal interstitial fibrosis to identify fibrotic areas for the assessment of PXDN expression. Therefore, the findings should not be generalized to broader ECM remodeling patterns or interpreted as evidence of a specific PXDN–COL3 interaction. Finally, although strong correlations were observed between PXDN and ECM remodeling markers, mechanistic links remain speculative. Future studies should address these limitations through larger, multicenter cohorts, longitudinal designs, inclusion of additional kidney diseases, broader fibrosis characterization, and experimental models to clarify the causal role of PXDN in fibrotic remodeling and its potential as a biomarker or therapeutic target in IgAN and CKD.

In summary, PXDN expression displays compartment-specific alterations in IgAN, with reduced tubular expression and increased expression in glomeruli and interstitium associated with fibrosis and declining kidney function. These findings support a potential association between PXDN and pathological remodeling processes in IgAN and identify PXDN as a candidate biomarker warranting further investigation. Although the functional contribution of PXDN to disease progression remains to be established, its involvement in ECM remodeling pathways suggests that it may also represent a potentially druggable target [25]. Future experimental studies are needed to determine whether modulation of PXDN activity could attenuate fibrotic remodeling and slow CKD progression.

## Supplementary Information

Below is the link to the electronic supplementary material.

Supplementary Material 1

Supplementary Material 2

Supplementary Material 3

Supplementary Material 4

Supplementary Material 5

Supplementary Material 6

Supplementary Material 7

Supplementary Material 8

## Data Availability

The datasets used during the current study will be available from the corresponding author upon request.
