# Supplementary material for: Peroxidasin expression patterns predict fibrosis and kidney function decline in IgA nephropathy
Source: Clin Exp Nephrol. 2026 Jul 27;30(11):1547–58. doi: 10.1007/s10157-026-02912-y (PMC13669128; doi:10.1007/s10157-026-02912-y)
Supplement: Supplementary file 1 [file 10157_2026_2912_MOESM6_ESM.docx]

**Supplementary Figure 1. Workflow of image processing and semantic segmentation of the kidney biopsies.**

After image acquisition [a], the different kidney compartments were manually segmented in unstained brightfield images using the QuPath software [b], allowing semantic segmentation of the kidney biopsy sample [c] — color codes: blue, glomeruli; pink, tubules; orange, vessels; green, interstitium. To distinguish the proximal tubules (CD15 positive) from the distal tubules (CD15 negative), annotations were transferred into CD15-labelled fluorescent images. The final annotations were then moved into peroxidasin (PXDN)-labelled fluorescent images and PXDN fluorescence intensity in the different kidney compartments was retrieved using ImageJ software.

**Supplementary Figure 2. Relationship between the number of segmented glomeruli per biopsy and the coefficient of variation (CV) of peroxidasin (PXDN) expression in glomeruli from patients with IgA nephropathy.**

Each point represents an individual biopsy sample with ≥2 segmented glomeruli (n = 17). The CV of PXDN expression across segmented glomeruli is plotted against the number of glomeruli analyzed in that biopsy. All samples showed CV values below 10%, indicating low intra-biopsy variability. Overall, the plot indicates that PXDN expression measurements are relatively stable across glomeruli, and increasing the number of segmented glomeruli does not change variability. CV = 100 x (standard deviation/mean).

**Supplementary Figure 3. Representative photomicrographs of the comparative expression of collagen type III (Col III) and collagen type IV (Col IV), in areas with (w/) or without (w/o) sclerotic/fibrotic lesions from IgA nephropathy kidney biopsy specimens.**

Brightfield images of biopsy specimens [a1, b1] and corresponding fluorescence images of Col III [a2, b2] and Col IV labelling [a3, b3], followed by overlay of the fluorescence images [a4, b4]. Details of the histology and staining methods are provided in Materials and Methods. The secondary antibodies used for the immunofluorescence staining were either [a2, b2] the Donkey Anti-Rabbit IgG (H+L) Highly Cross-Adsorbed Secondary Antibody, Alexa Fluor™ Plus 647 (Invitrogen, Thermo Fisher Scientific; Waltham, MA, USA; catalog # A32795), or [a3, b3] Donkey Anti-Mouse IgG (H+L) Highly Cross-Adsorbed Secondary Antibody, Alexa Fluor™ Plus 568 (Invitrogen, Thermo Fisher Scientific; Waltham, MA, USA; catalog # A10037). No non-specific fluorescence labeling was observed in control samples when the secondary antibody was used in the absence of the primary rabbit anti-PXDN antibody (data not shown). The brightfield and fluorescence images were acquired in a NanoZoomer S60 Digital Slide Scanner (Hamamatsu Photonics; Hamamatsu, Japan). Scale bar: 50 µm. Color staining codes: Col III, red; Col IV, yellow. *Additional abbreviation* *‒ IgA, immunoglobulin A.*

**Supplementary Figure 4. Comparative analyses of Sirius Red (SR) binding in non-fibrotic vs fibrotic areas in IgA nephropathy (IgAN) kidney biopsy specimens (n = 20; median age = 49.4 years).**

Brightfield images of SR staining of non-lesioned and fibrotic areas [a] and corresponding quantification of percentage of SR red pigment binding [b]. Details of the histology and staining methods are provided in Materials and Methods. The brightfield images were acquired in a NanoZoomer S60 Digital Slide Scanner (Hamamatsu Photonics; Hamamatsu, Japan). Scale bar: 50 µm. Statistical analysis was performed with GraphPad Prism® software paired T-test. The SR-stained area was detected by color threshold in Qupath, divided by the total area of the section, and multiplied by 100 to obtain the percentage. *Additional abbreviation* *‒ IgA, immunoglobulin A.*

**Supplementary Figure 5. Quantitative comparison of peroxidasin (PXDN) expression across histological compartments in IgA nephropathy (IgAN) kidney biopsy specimens (n = 19; median age = 49 years), stratified by semiquantitative histological assessment of glomerular IgA deposition (+, ++ and +++).**

All statistical analyses for the comparison of IgA deposition classification (+, ++ and +++) and the quantification of PXDN expression in the areas comprising the distal tubules and interstitium were performed with GraphPad Prism® software using Kruskal-Wallis and Dunn's multiple comparisons test (n_+_ = 2; n_++_ = 13; n_+++_ = 4). Intensity/area values were obtained by dividing the integrated density by the total area of the compartment. *Additional abbreviations ‒ a.u., arbitrary units; IgA, immunoglobulin A.*
