# Supplementary material for: Peroxidasin expression patterns predict fibrosis and kidney function decline in IgA nephropathy
Source: Clin Exp Nephrol. 2026 Jul 27;30(11):1547–58. doi: 10.1007/s10157-026-02912-y (PMC13669128; doi:10.1007/s10157-026-02912-y)
Supplement: Supplementary file 2 [file 10157_2026_2912_MOESM7_ESM.docx]

| **Supplementary Table 1.** Kidney segmentation reproducibility. | | |
| --- | --- | --- |
| **Annotated Structures** | **Inter-annotator Agreement** | |
|  | **Dice Similarity Coefficient (Mean ± SD)** | **Intersection-over-Union (Mean ± SD)** |
| Glomeruli | 0.94 ± 0.04 | 0.90 ± 0.07 |
| Tubules | 0.95 ± 0.02 | 0.90 ± 0.03 |
| Interstitium | 0.76 ± 0.12 | 0.63 ± 0.15 |
| Vessels | 0.84 ± 0.16 | 0.74 ± 0.18 |
| Inter-annotator reproducibility for segmentation of glomeruli, tubules, vessels, and interstitium was assessed on a random subset of 79 image tiles (512 × 512 pixels) independently segmented by two annotators. Agreement was quantified using the Dice Similarity Coefficient (DSC) and Intersection-over-Union and the agreement metrics reached a plateau at 70 image tiles, with no appreciable improvement observed thereafter.  Values are presented as mean ± SD. Statistical analyses were performed using R version 4.6.0 and RStudio version 2026.05.0. The segmentation masks, R scripts, and detailed instructions required to reproduce the analysis have been made publicly available through a GitHub repository and archived in Zenodo {Brandao I. KidneySegmentValidation. https://doi.org/10.5281/zenodo.20430981}. | | |
