# Supplementary material for: Peroxidasin expression patterns predict fibrosis and kidney function decline in IgA nephropathy
Source: Clin Exp Nephrol. 2026 Jul 27;30(11):1547–58. doi: 10.1007/s10157-026-02912-y (PMC13669128; doi:10.1007/s10157-026-02912-y)
Supplement: Supplementary file 3 [file 10157_2026_2912_MOESM8_ESM.docx]

| **Supplementary Table 2.** Correlation of PXDN expression with COL3 and COL4 expression across lesioned and non-lesioned kidney compartments. | | |
| --- | --- | --- |
| **Log PXDN Intensity/Area [a.u./μm^2^]** | **Spearman’s Correlation (ρ / *p-value*)** | |
|  | COL3 expression [a.u./μm^2^] | COL4 expression [a.u./μm^2^] |
| Glomeruli | 0.321 / 0.50 | 0.321 / 0.50 |
| Tubules | 0.714 / 0.09 | -0.036 / 0.96 |
| Interstitium | 0.536 / 0.24 | -0.07 / 0.91 |
| Sclerotic Glomeruli | 0.821 / 0.03* | 0.107 / 0.84 |
| Atrophic Tubules | 0.500 / 0.27 | -0.357 / 0.44 |
| Fibrotic Interstitium | 0.929 / 0.007* | -0.179 / 0.7131 |
| Spearman’s rank correlation (ρ) was used because normality assumptions were not met.  COL3, collagen type III; COL4, collagen type IV. *Statistically significant correlation. | | |
|  | | |
